# Explore the Role of Abeta in Axonal Trafficking Deficits Induced by Alpha Synuclein in Parkinson Disease Mouse Model

**DOI:** 10.1093/geroni/igab046.3694

**Published:** 2021-12-17

**Authors:** Alfredo Castro

**Affiliations:** UCSD MADURA R25 Advancing Diversity in Aging Research Program, La Jolla, California, United States

## Abstract

Alpha synuclein (ASYN) is a neuronal protein that is observed in significant amounts in the brain and is encoded for by the SNCA gene, it functions as a regulator for the trafficking of synaptic vesicles. It has been noted that the buildup of alpha synuclein has been found in the form of Lewy bodies in studies involving patients with Parkinson’s diseases (PD). Gathering an understanding for the manner in which alpha synuclein affects the synaptic structure and the movement of axonal trafficking will help further our understanding towards the formation of Lewy bodies. Experimenting with the way in which ASYN affected the intervention of Abeta was important, to see the toxicity of Abeta in axonal trafficking. The PD and SynKO mouse models treated with Abeta both showed an effect on the anterograde moving speed of both the PD and SynKO neurons. Synaptic formation was examined, and it was found that ASYN had a large negative influence on the synapse formation in PD neurons. This was due to the significantly reduced colocalization that was found in the treated neurons. It was confirmed that ASYN caused neuronal atrophy through the over expression of GFP-ASYNWT wild type or the GFP-ASYNA53T. Comprehending ASYN effect on the axonal trafficking and the synaptic structure of PD neurons can help understand the mechanism that may be present which possibly stimulates Alzheimer’s Disease in PD patients.

